# The Efficiency of the Human CD8+ T Cell Response: How Should We Quantify It, What Determines It, and Does It Matter?

**DOI:** 10.1371/journal.pcbi.1002381

**Published:** 2012-02-23

**Authors:** Marjet Elemans, Nafisa-Katrin Seich al Basatena, Becca Asquith

**Affiliations:** Section of Immunology, Imperial College School of Medicine, London, United Kingdom; Emory University, United States of America

## Abstract

Multidisciplinary techniques, in particular the combination of theoretical and experimental immunology, can address questions about human immunity that cannot be answered by other means. From the turnover of virus-infected cells in vivo, to rates of thymic production and HLA class I epitope prediction, theoretical techniques provide a unique insight to supplement experimental approaches. Here we present our opinion, with examples, of some of the ways in which mathematics has contributed in our field of interest: the efficiency of the human CD8+ T cell response to persistent viruses.

This is an “Editors' Outlook” article for *PLoS Computational Biology*

## Introduction

**Figure 1:**
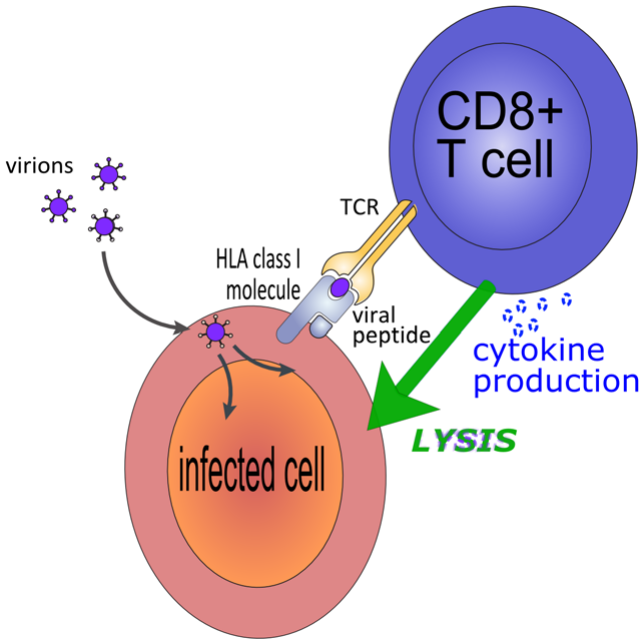
CD8+ T cells recognise virally infected cells via their T cell receptor (TCR). The TCR binds complexes of viral peptides and HLA class I molecules at the surface of virus-infected cells. Following CD8+ T cell recognition of the infected cell, the CD8+ T cell effector mechanisms are triggered. CD8+ effector mechanisms can be lytic (killing of the infected cell) and/or non-lytic (secretion of cytokines such as IFNg and TNFa which reduce the probability of cell infection and viral production).

We all carry lifelong, persistent viral infections. More than 70% of adults are infected with Epstein Barr Virus (EBV), 80%–90% with Cytomegalovirus, and >90% with Varicella Zoster Virus. Worldwide, 170 million people are infected with Hepatitis C virus (HCV) and 33 million with Human Immunodeficiency Virus (HIV-1). CD8+ T cells are thought to play an important role in controlling these persistent viral infections. CD8+ T cells recognise viral antigen in the context of HLA class I molecules (Figure 1) and are thus activated to kill virus-infected cells via perforin/granzyme secretion or the Fas/FasL pathway and to secrete antiviral cytokines. Some persistent viruses such as EBV or Herpes Simplex Virus adopt a strategy of latency to evade the host immune response. These viruses are characterised by low or intermittent levels of antigen expression and a corresponding low level of CD8+ T cell activation. Their mechanism of survival is clear. However, other viruses such as HIV-1, HCV, and human T cell leukemia virus (HTLV-1) replicate persistently and induce chronic activation of the virus-specific CD8+ T cell response. Yet despite continuous exposure to this CD8+ T cell response, which is often large (up to 30% of CD8+ T cells can be specific for HIV-1 or HTLV-1) and typically has immediate effector function ex vivo, the CD8+ T cell response fails to eradicate these viruses. Why? In order to understand why the CD8+ T cell response fails and how we can improve it, we need to be able to measure the impact of the CD8+ T cell response in vivo, understand what constitutes an efficient response, and more controversially, tackle the in vivo relevance of the CD8+ T cell response. We maintain that, for humans, none of these questions can be answered with experiment or mathematics alone. Here we review recent interdisciplinary work to address these questions in the context of persistent viruses in humans.

## Quantification of CD8+ T Cell Efficiency

We define the efficiency of the CD8+ T cell response as the rate at which infected cells are killed or new infections are prevented; that is, efficiency measures the antiviral pressure exerted by the whole virus-specific CD8+ T cell population. Quantifying antiviral pressure in vivo is impossible without the use of mathematics.

Mathematical modelling of experimental data allows us to estimate parameters, such as the rate of CD8+ T cell killing, that are not explicitly measured experimentally but which determine the data. Two types of experimental data have been used to quantify CD8+ T cell efficiency in humans in vivo, both in the context of HIV-1 infection. The first study, by Wick et al. [1], analysed a clinical procedure in which CD8+ T cells were isolated from three HIV-1-infected patients, expanded in vitro with HIV-1 peptides, and then reinfused. Wick et al. fitted a model to the subsequent productively infected cell dynamics and estimated that HIV-1-specific CD8+ T cells killed productively infected cells at a median rate of 0.129 µl cell^−1^ day^−1^. There are approximately 50–65 HIV-specific CD8+ T cells per µl of plasma ([2] gives median CD8 count = 500 cells µl^−1^, average frequency of HIV-1-specific CD8+ T cells = 10%; i.e., a specific count of 50 specific CD8 µl^−1^; [3] gives median CD8 count = 860 cells µl^−1^, average frequency of HIV-1-specific CD8+ T cells = 7.5%; i.e., a specific count of 65 specific CD8 µl^−1^). So Wick's estimate is equivalent to a lytic efficiency of 6.5–8.4 day^−1^. This estimate of CD8+ T cell killing is an order of magnitude higher than the *total* death rate attributable to *all* causes (including CD8+ T cell killing, activation induced cell death, and cytopathic effect of HIV-1), which has been estimated at 0.7–1 day^−1^ [4], [5]. The apparent overestimate of CD8+ T cell killing may be explained by biological factors; for example, the infused expanded CD8+ T cells could be more efficient than naturally occurring CD8+ T cells. Alternatively, the problem may lie in the model assumptions. For instance, it was assumed that all antiviral effects were exerted via lysis (i.e., reduction in infected cells due to non-lytic factors would have been attributed to lysis, Box 1), and many of the fixed parameters in the model were poorly known at the time of the study. Nevertheless, this represents the first estimate of the lytic potential of human CD8+ T cells in vivo.

**Box 1. Models of Lytic and Non-Lytic CD8+ T Cell Mechanisms**

A model in which CD8+ T cells are assumed to kill infected cells typically has the following form:
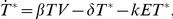
where T^*^ is the number of virus-infected cells, V is viral load, β is the infection rate, T is the number of uninfected target cells, E is the number of virus-specific CD8+ T cells, k is the rate of CD8+ T cell killing, and δ is the (CD8+ T cell-independent) death rate of infected cells.

Non-lytic mechanisms can decrease the rate at which uninfected CD4+ T cells become infected or the rate of viral production. We have modelled the impact of CD8+ T cells on the rate of infection [19], [21] by describing the infection rate as a function of the size of the CD8+ T cell population:
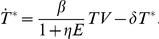
A non-lytic effect of CD8+ T cells on viral production rate can be described in a similar way [19], [21].

Most models of the CD8+ T cell response assume that CD8+ T cells lyse their targets. If in fact CD8+ T cells act mainly via non-lytic mechanisms, then this will change the model conclusions and may explain some of the discrepancies in the literature. For instance, if most CD8+ T cell control is mediated via non-lytic mechanisms, this may explain why Wick's estimates of CD8+ T cell killing are in excess of the total death rate of infected cells; that is, control of infected cells may have been misattributed to killing of infected cells, leading to an overestimate of their death rate. How non-lytic mechanisms will affect death rates inferred from HIV-1 escape from CD8+ T cells is unclear; we are currently investigating this question.

In the second study, the rate of CD8+ T cell killing was estimated by analysing HIV-1 escape variants [6], [7]. Briefly, the difference in growth rate in vivo between a viral variant which has escaped the CD8+ T cell response and the wild type reflects the difference in rate of CD8+ T cell killing of the two strains offset by any fitness cost incurred by the escape variant. Therefore the rate of CD8+ T cell killing (attributable to a single response) can be estimated by quantifying the rate of escape and the fitness cost. The rate of escape and fitness cost can be quantified by fitting a simple mathematical model to longitudinal viral sequence data [6]. This work indicated that, in late primary/chronic infection, CD8+ T cell efficiency was unexpectedly low (0.1–0.2 d^−1^), and thus CD8+ T cell killing was responsible for only 10%–20% of infected cell death. This estimate is lower than, and therefore compatible with, the total death rate of productively infected cells of 0.7–1 d^−1^, but the lack of independent quantitative approaches makes it difficult to corroborate this estimate. If it is true that CD8+ T cells are only responsible for a minority of infected cell death, this would explain the failure to see a decrease in infected cell death when the CD8+ T cell response is weak such as in individuals with low CD4+ T cell count [8] or following CD8+ T cell depletion in simian immunodeficiency virus (SIV)-infected macaques (a model of HIV-1 infection) [9], [10]. Furthermore, a large amount of cell death that is not attributable to direct CD8+ T cell killing is consistent with the high death rate of uninfected cells which has been observed in HIV-1 infection [11], [12]. Finally, a low estimate of CD8+ T cell killing in chronic infection should perhaps not be totally unexpected as persistent viral infection has been associated with reduced CD8+ T cell function and exhaustion, particularly in the context of HIV-1 infection [13]–[16]. Consistent with this, it was shown that escape was significantly more rapid in late primary than in chronic HIV-1 infection [6], an observation that was confirmed and extended by subsequent work in very early HIV-1 infection [17]. The cause of this difference remains unknown [18].

In the absence of other studies to quantify the efficiency of human CD8+ T cells in vivo, it is necessary to turn to animal models, where a wider range of approaches are possible, to corroborate the estimates in humans. In chronic SIV infection, estimates of total CD8+ T cell efficiency derived from CD8+ depletion experiments (median 0.3–0.4 d^−1^ [19]) and from modelling longitudinal data using the approach of Regoes et al. [20] (median 0.76 d^−1^ [21]) are similar to estimates from modelling SIV escape in chronic infection (approximately 0.8 d^−1^) [22], [23], lending credence to the quantification of efficiency from viral escape. Interestingly, both in chronic [6], [22], [23] and primary infection [6], [17], [22]–[25], the rate of CD8+ T cell killing was substantially higher in macaques than in humans [22]. Suggestions to the contrary [23] failed to take into account differences in fitness costs between wild type and escape variant virus. In LCMV-infected mice, the rate of CD8+ T cell killing is 2–3 orders of magnitude higher than in humans or macaques in both acute and chronic infection [26]–[29]; further studies are urgently needed to understand whether these differences are genuine inter-species differences, differences between viruses, or simply differences in experimental and theoretical techniques. For an excellent, considerably more comprehensive, review of work to quantify CD8+ T cell efficiency, see Regoes et al. [30].

## Determinants of CD8+ T Cell Efficiency

Identifying the determinants of an efficient CD8+ T cell response in humans is non-trivial. Measurements of CD8+ T cell frequency, phenotype, function, and specificity are informative, but because antigen load influences each of these factors, it can be difficult to ascertain if a particular immune profile is the cause or effect of good pathogen control [31]–[33]. This is exemplified by one ubiquitous method to determine the strength of the CD8+ T cell response: measurement of the frequency of virus-specific cells (by, e.g., tetramer or IFNg ELISpot) with the assumption that a large response is a strong, efficient one. In a cross-sectional cohort of HIV-1-infected individuals, a negative correlation between the frequency of tetramer-positive CD8+ T cells and plasma viral load was observed; it was concluded from this that CD8+ T cells controlled HIV-1 viremia [34]. However, theoretical work has shown that inferring CD8+ T cell control from CD8+ T cell frequency is problematic because of feedback between viral load and CD8+ T cell frequency [35], [36]; that is, the frequency is both the cause and effect of the viral load. This concern has been borne out by subsequent longitudinal studies in which the magnitude of the HIV-1-specific CD8+ T cell response failed to predict viral load or survival time [37]–[39] and elite control of HIV infection was associated with the lowest breadth and frequency of HIV-specific CD8+ T cells [40]. We conclude that purely experimental descriptors of the CD8+ T cell response tell us about the correlates, but not the determinants, of good pathogen control.

An alternative approach is to focus on host genetic determinants of CD8+ T cell efficiency (as here the direction of causality is unequivocal) and use mathematical methods to understand why certain genes are protective or detrimental. This approach was applied very effectively by Borghans et al. [41], who found that HLA class I alleles that are significantly associated with good control of HIV-1 infection (HLA-B*57:01, B*58:01, and B*27:05) showed a preference for presenting peptides from the viral Gag p24 protein when compared with alleles associated with poor control (B*35:03 and B*53:01). Borghans et al. conclude that Gag-specific CD8+ T cells are protective. It is therefore surprising that Vider-Shalit et al. found that over the course of the HIV epidemic, in contrast to other HIV proteins, Gag is evolving to *gain* epitopes [42]. They hypothesised that Gag-specific CD8+ T cell responses were inefficient and Gag immunogenicity had evolved as a decoy to reduce the CD8+ T cell response against proteins expressed early in the viral life cycle which may be more effective targets [43]. However, this result was not reproduced by Schmid et al., who showed that the number of Gag epitopes (in common with all HIV-1 CD8+ T epitopes) has remained remarkably constant in the population over time [44]. The two main differences between the work of Schmid and Vider-Shalit are the choice of epitope prediction software and the dating applied to the sequences. Schmid et al. used NetCTL and MHCpathway, which were the best performing algorithms at the time [45]; the software used by Vider-Shalit has not been benchmarked. Schmid dated sequences by time of sampling, and Vider-Shalit inferred dates from levels on a phylogentic tree. In principal it should be straightforward to investigate these discrepancies and arrive at a definitive conclusion.

In a related study, MacNamara et al. used HLA class I epitope prediction software [45]–[48] to investigate why, in HTLV-1 infection, some individuals remained healthy, asymptomatic carriers of the virus and others developed the inflammatory disease HAM/TSP. In a cohort of HTLV-1-infected participants, they showed that individuals with HLA class I alleles that were predicted to strongly bind the HTLV-1 protein HBZ had a lower proviral load and were more likely to be asymptomatic [49]. They also showed that CD8+ T cell effectiveness is determined by protein specificity and produced a ranked list of the proteins targeted by the most protective CD8+ T cell response through to the least protective CD8+ T cell response: useful information for vaccine design. They concluded that CD8+ cells specific to HBZ, not the immunodominant protein Tax, are the most protective. This challenges the prevailing assumption that the immunodominant response is the most important.

More generally, Rao et al. have studied the differences between HLA-A and HLA-B molecules [50]. The HLA-B locus is the most polymorphic and the most rapidly evolving of the three major HLA class I loci, and it has also been suggested that CD8+ T cell responses restricted by HLA-B alleles are more likely to be protective [51]. Using a bioinformatic approach Rao et al. found that HLA-B epitopes had significantly weaker binding affinities than HLA-A epitopes and were less diverse [50]. These observations ran counter to many people's assumptions and have triggered further investigations.

The HLA class I genes are not the only genetic determinants of CD8+ T cell efficiency that have been subjected to a theoretical approach. Genes coding for the killer immunoglobulin-like receptors (KIRs) have also been analysed. KIRs are a family of receptors that are expressed predominantly on natural killer (NK) cells but also on a subset of T cells [52], [53]. KIRs bind HLA class I molecules and have both activating and inhibitory forms [54]. KIRs are rapidly evolving, and even amongst primates, only our nearest relatives possess the same KIR-HLA ligand pairs as us. In mice, the functional homologue of KIRs are the Ly49 receptors. These receptors are quite distinct from KIRs in structure and patterns of expression [55]. Lack of an accurate animal model has hindered work on the role of KIRs in adaptive immunity, making a theoretical approach particularly beneficial. Seich al Basatena et al. tested the hypothesis that KIR background determines the efficiency of the CD8+ T cell response [56]. They investigated two chronic viral infections—HCV and HTLV-1—using cohorts of infected individuals. They selected HLA class I molecules previously described to be significantly associated with clinical outcome (HLA-B*57 in HCV [57]–[59]; C*08, A*02, and B*54 in HTLV-1 [60], [61]), stratified the cohorts by KIR genotype, and then re-examined the HLA associations in each stratum. They found that one particular inhibitory KIR gene, namely *KIR2DL2*, consistently enhanced HLA class I associations. That is, associations between outcome and HLA class I alleles were strong when *KIR2DL2* was present but weak when *KIR2DL2* was absent [56]. For instance, in HCV infection, amongst *KIR2DL2*-homozygotes, individuals with HLA-B*57 were 5 times more likely to spontaneously clear the virus than individuals without B*57; if they failed to clear they had a viral load that was reduced by 6.5 logs. In *KIR2DL2* heterozygote individuals, B*57 was still protective but the degree of protection afforded was reduced: individuals with B*57 were now twice as likely to spontaneously clear the virus; if they failed to clear, viral load was reduced by 4 logs. In contrast, in *KIR2DL2*-negative individuals, B*57 had no significant effect on clearance or viral load. It appears that B*57, a well-described protective allele, only efficiently protects if *KIR2DL2* is also present. If an additional gene needs to be present for an HLA class I molecule to be effective, then this could explain why the protective or detrimental effects of particular HLA alleles are not always manifest. Applying this reasoning to HTLV-1 it was possible to explain two long-standing paradoxes in the field: why C*08 doesn't reduce proviral load in HAM/TSP patients and why B*54 isn't associated with increased proviral load in asymptomatic carriers.

**Figure 2:**
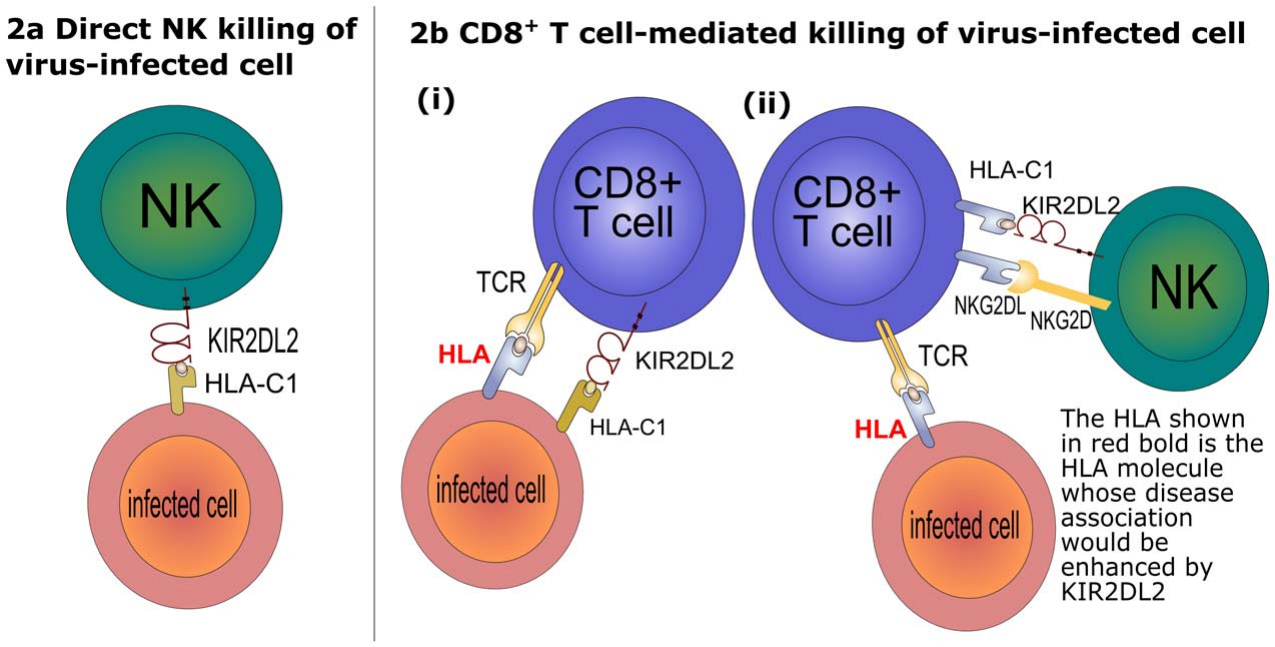
Proposed mechanism to explain inhibitory KIR enhancement of HLA class I associations. KIR-HLA associations are typically attributed to direct NK action (a). However, Seich al Basatena's observations do not appear to be compatible with direct NK killing for four reasons. Firstly, most of the HLA molecules which were enhanced do not bind KIR2DL2. Secondly, both protective and detrimental HLA associations were enhanced. Thirdly, KIR2DL2 with or without its C1 ligand (i.e., not in the context of a particular protective or detrimental HLA allele) had no effect on any outcome for HCV or HTLV-1. And finally, the protective effect of binding HBZ was enhanced, and NK cells display some peptide specificity, but such marked protein specificity is more reminiscent of T cells [56]. Instead they hypothesise that the downstream effectors are CD8^+^ T cells (b). They postulate that KIR2DL2 enhances CD8^+^ T cell responses by increasing the survival of memory CD8+ T cells. Either because (i) inhibitory KIR on CD8^+^ T cells are associated with reduced activation induced cell death and protection from exhaustion [77]–[79] or (ii) inhibitory KIR on NK cells reduce NK-killing of activated CD8^+^ T cells [80], [81].

Many associations between disease and pairs of KIR-HLA genes have been reported [62]–[66]. In each case the KIR-HLA pair consisted of a KIR with its HLA ligand and the effect was attributed to direct NK killing. What Seich al Basatena et al. observed is quite different. They found that associations between HLA class I molecules and disease outcome are weak in the absence of *KIR2DL2* but are enhanced in the presence of *KIR2DL2*. This is true for multiple HLA-A, B, and C alleles, most of which do not bind KIR2DL2. They saw this effect in two different virus infections and for both protective and detrimental HLA associations. In contrast, KIR2DL2 with its HLA-C1 ligand (not in the context of protective or detrimental HLA molecules) has no detectable impact on any measure for either virus [56]. It is hard to reconcile these observations with direct NK killing (Figure 2a). Instead they hypothesised that KIR2DL2, a receptor typically associated with innate immunity, is enhancing the effectiveness of the CD8+ T cell response by increasing CD8+ T cell survival (Figure 2b). This is an example in which theory has suggested new biology: a novel role for an “innate” receptor in adaptive immunity.

Temporal and spatial factors—for example, how often does a CD8+ T cell meet infected cells bearing a sufficient density of cognate antigen—will also impact of the effectiveness of CD8+ T-cell-mediated control. Mathematical modelling, using cellular automata [67], cellular Potts models [68], and the multicompartment models, exemplified by the work of Textor et al. [69], is contributing to the identification of the physical determinants of CD8+ T cell efficiency, but this work is beyond the scope of the current review.

## Does CD8+ T Cell Efficiency Matter?

One of the hardest, but perhaps the most important, question to answer is, What is the impact of CD8+ T cell efficiency on clinical outcome and to what extent do between-individual differences in clinical outcome and viral load reflect differences in CD8+ T cell efficiency? The two strongest arguments to suggest that CD8+ T cells matter in chronic infections of humans are (i) the observation that certain HLA class I alleles are significantly associated with clinical outcome and (ii) that genetically variable viruses like HIV-1 and HCV evolve mutations that allow them to evade the CD8+ T cell response. These observations tell us that CD8+ T cells matter but, alone, do not tell us how much they matter. Interdisciplinary studies have taken these observations and used them to quantify the in vivo relevance of CD8+ T cells.

**Figure 3:**
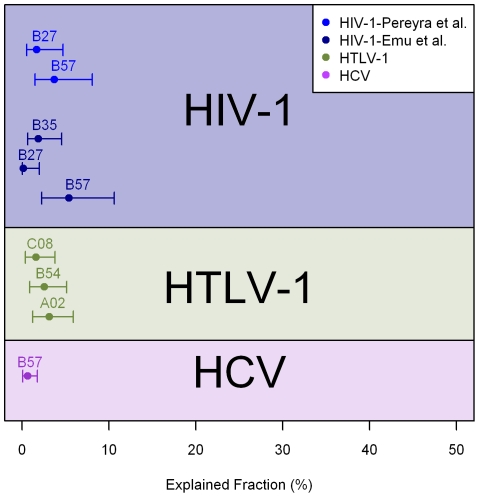
The variation in outcome that can be explained by individual HLA class I alleles in HIV-1, HTLV-1 and HCV infections. The explained fraction (EF) was calculated following Nelson [82]; data for HCV and HTLV-1 were taken from [56], and data for HIV-1 were taken from [40], [83]. 95% confidence intervals were estimated by bootstrapping the data 5,000 times, trimming the 5% extremes, and then calculating the range in which 95% of the remaining data lay. Due to linkage between the HLA alleles, the EF is not additive; so for instance, in HTLV-1 infection, the three alleles HLA-A*02, C*08, and B*54 together only explain 6.6% of the outcome. The outcomes explained are HCV: spontaneous clearance v persistence; HTLV-1: asymptomatic carriage v HAM/TSP; HIV-1: elite control v viremic control v progression.

- ***HLA Associations***. Surprisingly, although associations between particular HLA class I molecules and clinical outcome are highly significant, the fraction of variation in outcome that can be explained by HLA alleles is rather small (Figure 3), though this does increase by a factor of 2–4 in *KIR2DL2*+ individuals. Clearly, HLA associations tell us about differences in HLA molecules, so a small explained fraction does not necessarily mean CD8+ T cell responses are unimportant; it could be that all CD8+ T cell responses are rather similar regardless of their restriction. However, it does caution us that we should not assume that the CD8+ T cell response is important just because HLA associations are highly significant. Furthermore, it implies that the majority of between-individual variation in clinical outcome is unlikely to be due to differences in HLA class I genotype.
- ***Viral Escape***. Kadolsky et al. used two independent computational methods to quantify the impact of escape from CD8+ T cells in chronic HIV-1 infection: simulation of the emergence of HIV-1 escape variants and statistical analysis of a cross-sectional cohort [70]. Both methods estimated that escape at a single epitope increased log viral load by 0.051–0.11 copies.ml^−1^. The number of escape events accounted for approximately 6% of the viral load variation in the cohort [70]. Thus, escape from CD8+ T cells has a measurable, statistically significant impact on viral load, but the biological impact is modest. This small increase in viral load may reflect a high fitness cost of the escape variant virus (though estimates of fitness cost are typically quite low [6], [25]), or it may indicate that, in chronic HIV-1 infection, the weakened CD8+ T cell response is exerting poor control. Again this study implies that differences in CD8+ T cell escape cannot explain the substantial inter-individual variations in HIV viral load.

## Mechanism of Action of CD8+ T Cells

Perhaps the most immediate demonstration of the importance of CD8+ T cell is the dramatic rise in viral load following their depletion [71], [72]. Klatt et al. [9] and Wong et al. [73] applied the pioneering mathematical method of Perelson et al. and Nowak et al. [74], [75] to estimate the lifespan of productively infected cells in CD8+ cell-depleted macaques and control macaques with an intact CD8+ T cell response. Unexpectedly, they found no difference in cell lifespan. The authors concluded that CD8+ T cells don't play a major role in decreasing the lifespan of productively infected cells. They proposed instead that CD8+ T cells control viremia via a non-lytic mechanism.

Elemans et al. [19] extended this study. They found that the measurements of infected cell lifespan of Klatt et al. and Wong et al. were not accurate enough to detect the expected increase in lifespan following depletion, implying that the experimental results could not exclude the possibility that all CD8+ T cell control was via lysis. However, on fitting an extensive set of lytic and non-lytic models to the experimental data (including models from [8], [76]), they found that a non-lytic model in which CD8+ T cells prevent new cells from becoming infected predicted the data significantly better than other models [19]. They conclude that the total rate at which infected cells are killed or new infections are prevented was 0.3–0.4 d^−1^ and that most of this control was via nonlytic mechanisms. These results are compatible with earlier findings that most of the death of productively infected cells is CD8+ T-cell-independent [6], [21].

## Future Directions

Mathematical modelling of experimental data can provide a unique angle to help understand human immunity. However, mathematics continues to raise as many questions as it answers. Why is CD8+ T cell killing so much more effective in mice than men? Why does CD8+ T cell efficiency decline in HIV-1 infection, and how does KIR2DL2 enhance CD8+ T cell efficiency? These are just some of the questions raised by a quantitative approach that need to be addressed.

**Authors' Biographies**

**Marjet Elemans** is a computational biologist at Imperial College London. She has a broad interest in the basic biology of immune cell function and the population dynamics of the immune response. She has recently worked on modelling the immune response against viral infections, in particular the CD8+ T cell response against HIV-1 infection.

**Nafisa-Katrin Seich al Basatena** is a Wellcome Trust PhD student in the Department of Immunology. As an undergraduate she studied Applied Mathematics and Physical Sciences at the National Technical University of Athens followed by an MSc in Mathematical Modelling and Scientific Computing at Oxford University and an MSc in Bioinformatics and Theoretical Systems Biology at Imperial College. Her research focuses on genetic factors involved in viral infections and mathematical modelling of T cell immune responses in vivo.

**Becca Asquith** is a Senior Lecturer in Within Host Dynamics in the Department of Immunology at Imperial College London. Her initial training (to PhD) is in Theoretical Particle Physics. She works as a Mathematical Immunologist with a particular interest in the human CD8+ T cell response and in vivo lymphocyte dynamics.

Our group webpage is: http://www.imperial.ac.uk/departmentofmedicine/theoreticalimmunology.
